# Fermentation and Storage Characteristics of “Fuji” Apple Juice Using *Lactobacillus acidophilus, Lactobacillus casei* and *Lactobacillus plantarum*: Microbial Growth, Metabolism of Bioactives and *in vitro* Bioactivities

**DOI:** 10.3389/fnut.2022.833906

**Published:** 2022-02-09

**Authors:** Jie Yang, Yue Sun, Tengqi Gao, Yue Wu, Hao Sun, Qingzheng Zhu, Chunsheng Liu, Chuang Zhou, Yongbin Han, Yang Tao

**Affiliations:** ^1^Jiangsu Key Laboratory of Marine Bioresources and Environment/Jiangsu Key Laboratory of Marine Biotechnology, Jiangsu Ocean University, Lianyungang, China; ^2^Co-Innovation Center of Jiangsu Marine Bio-industry Technology, Jiangsu Ocean University, Lianyungang, China; ^3^Sonochemistry Group, School of Chemistry, The University of Melbourne, Parkville, VIC, Australia; ^4^Department of Animal Husbandry and Veterinary Medicine, Jiangsu Vocational College of Agriculture and Forestry, Jurong, China; ^5^College of Food Science and Technology, Whole Grain Food Engineering Research Center, Nanjing Agricultural University, Nanjing, China

**Keywords:** lactic acid bacteria, apple juice, fermentation, physicochemical properties, bioactivities

## Abstract

Fruit juices have been widely used as the substrates for probiotic delivery in non-dairy products. In this study, three lactic acid bacteria (LAB) strains, including *Lactobacillus acidophilus, Lactobacillus casei* and *Lactobacillus plantarum*, were selected to ferment apple juice. During 72-h of fermentation, these LAB strains grew well in the apple juice with significant increases in viable cell counts (from 7.5 log CFU/mL to 8.3 log CFU/mL) and lactic acid content (from 0 to 4.2 g/L), and a reduction of pH value (from 5.5 to around 3.8). In addition, the antioxidant and antibacterial capacities of fermented apple juice *in vitro* were significantly improved through the phenolic and organic acid metabolisms. After storage at 4°C for 30 days, the total amino acid content of fermented apple juice was significantly increased, although the viable cell counts and total phenolic content were decreased (*p* < 0.05). Furthermore, the stored fermented apple juices still possessed antibacterial and *in vitro* antioxidant activities. Overall, all the selected LAB strains could be suitable for apple juice fermentation and can effectively improve their biological activities.

## Introduction

Probiotics are living microorganisms and potential functional foods, which have a positive impact on the human body by improving the microbial balance in the human intestine (1). Lactic acid bacteria (LAB) are important probiotics, and they are often used in yogurt and other dairy products. In recent years, the researches on fermented fruit and vegetable juices by lactic acid bacteria have gradually emerged. Meanwhile, the related products in the Chinese market are still limited, which could not meet the consumer's demand. After the fermentation of fruit and vegetable juices by LAB strains, their nutritional and functional values are improved, which not only gives fruit and vegetable juice a special taste and taste, but also extends their shelf life (2, 3).

Apple is one of the most consumed fruits in the world, especially in “fuji” and related varieties which are widely accepted by consumers because of its excellent sensory properties (4, 5). They are characterized by high sugar content and low acidity (6). With the improvement of living standards, consumers are more concerned about the freshness, originality, physicochemical properties, and nutritional values of food products including fruit juice (7). Apple juice has some positive impacts on human health due to its rich contents of polyphenols such as isoflavones, flavonoids and phenolic acids (8). Although fruit juices are rich in minerals, sugars and vitamins, they contain low amounts of free peptides and amino acids, which is adverse to the growth and metabolism of human intestinal microorganisms (9). To address this problem, LAB strains are widely used for the fermentation of fruit and vegetable substrates due to their excellent tolerability in acidic environments. In addition, the nutrients of apples are retained due to the fermentation by probiotics, resulting in unique flavor and efficacy (8). In the literature, Chen et al. (10) studied the effect of four LAB strains on the flavors of fermented apple juice. The results showed that the total acid concentration and microbial activity under the fermentation of lactic acid bacteria increased markedly, and new volatile compounds with important flavors were produced. Kwaw et al. (11) reported that the effects of lactic acid bacteria on the color, phenolics (such as anthocyanins) and antioxidant activity of mulberry juice. The results found that lactic acid fermentation can enrich the bioactive components in mulberry juice. Similarly, Vivek et al. (12) found an increase trend of antioxidant activity, total phenolic and anthocyanin contents in “Sohiong” juice fermented by *L. plantarum*, confirming the suitability and feasibility of developing a non-dairy based probiotic drinks from Sohiong juice. Kaprasob et al. (13) also reported an increase in phenolic compounds, vitamin C and other antioxidants in cashew apple juices fermented by *L. casei, L. plantarum* and *L. acidophilus*. In theory, by using the selected three lactic acid bacteria, apple juice can be processed into value-added products. However, there is still limited researches about the effect of LAB on the fermentation and preservation of apple juice.

In this study, three potential probiotics, *L. plantarum, L. acidophilus* and *L. casei*, were selected to ferment apple juice. The changes of organic acids, sugars, free amino acids, and phenolics during fermentation and storage (4°C for 30 days) were evaluated. Finally, this study can provide the basic knowledge of the biotransformation of relevant components in apples by LAB fermentation, and guide the development of fermented apple juice products with high nutritional value and safety.

## Materials and Methods

### Materials and Chemicals

Fresh apple was provided by Xuanwu Fruit Store, Nanjing, Jiangsu Province. Ethanol, acetonitrile, Folin-Ciocalteu reagent, organic acid standards (oxalate acid, pyruvic acid, malic acid, citric acid, succinic acid and lactic acid), phenolic acid standards (gallic acid, protocatechuic acid, catechin, procyanidin B_2_, chlorogenic acid, hydroxybenzoic acid, caffeic acid, ferulic acid, rutin and quercetin), amino acid standards (Asp, Thr, Ser, Glu, Gly, Ala, Cys, Val, Met, Ile, Leu, Tyr, Phe, Lys, His, Arg, Pro) and sugar standards (fructose, sorbitol, glucose and sucrose) were all bought from Yuanye Biotechnology Co., Ltd (Shanghai, China).

### Lactic Acid Bacteria Strains and Inoculum Preparation

Three commercial LAB strains *L. acidophilus* BNCC 185342, *L. casei* ATCC 393, *L. plantarum* BNCC 337796 were provided by Beijing Beina Chuanglian Biotechnology Research Institute (Beijing, China). All stains were activated in MRS broth (Bo Microbiology Technology Co., Ltd. Shanghai) at 37°C for 24 h to achieve a final concentration at 9.0 log CFU/mL.

### Preparation of Apple Juice and Fermentation

Fresh apples (“Red Fuji” apple from Xuanwu Fruit Store, Nanjing, Jiangsu Province) were washed, peeled, nucleated, and cut into small pieces. Next, 0.15% (w/w) ascorbic acid (also known as vitamin C) was added during crushing. Then, apple slurries were filtered through four layers of gauze and centrifuged at 6,000 × *g* for 20 min. The supernatant was collected, and the soluble solid content and pH were adjusted to 13% using glucose and 6.5 using 1N NaOH, separately.

Apple juice was sterilized at 110°C for 10 min before fermentation. After cooling to room temperature, 8 mL of inoculum containing various LAB strains were added into 400 mL of apple juice, respectively. The initial viable bacterial count in apple juice was about 7.5 log CFU/mL. Next, apple juice with different LAB strains were placed into an incubator to ferment for 72 h at 37°C. After that, fermented juices were immediately stored at 4^o^C for 30 days. Unfermented apple juice was selected as a control group. Samples were collected for analysis during fermentation at 0, 12, 24, 48, 72 h and storage at 10, 20, 30 d. Before the physicochemical analysis, the collected samples were centrifuged at 4°C and 12,000 × *g* for 15 min.

### Determination of Viable Cell and pH

The number of viable bacteria was determined by the plate counting method. Specifically, 1 mL fermented juices were diluted serially with sterile saline to 10^4^-10^10^ dilutions. Inoculation of 0.1 mL of the diluted samples were plated on the MRS agar. The plates were incubated at 37°C for 36–48 h. Plates containing 30–300 colonies were counted and recorded as log CFU/mL (14). The pH of fermented apple juice was monitored by a digital pH meter (PHS-3C, Shanghai INESA Scientific Instrument Co., Ltd, Shanghai, China).

### Determination of the Browning Index

The browning index of unfermented and fermented apple juice samples were measured referring to Tiwari et al. (15). 3 mL of centrifuged apple juice was thoroughly mixed with 3 mL of anhydrous ethanol, and then the mixture was centrifuged at 4°C and 12,000 × *g* for 15 min. The supernatant was collected, and its absorbance was measured at 420 nm in a spectrophotometer (LabTech UV9100, USA).

### Determination of Organic Acid Contents

Organic acids were determined by a LC-2010A system (Shimadzu Company, Japan) according to the previously reported method (16). The mobile phase is KH_2_PO_4_ (0.08 mol/L, pH 2.9). An Agilent TC-C18 chromatographic column (250 × 4.6 mm i.d., 5 mm) was applied for the separation of organic acids and it was maintained at 30°C. The injection volumes of the samples or standards were 20 μL, and the flow rate was 0.7 mL/min. Organic acids were detected using an ultraviolet detector (DAD) at 210 nm. The content of each organic acid was expressed as mg/L.

### Determination of Sugar Contents

The sugars were determined by a HPLC-ELSD system (Agilent Technologies, USA., Alltech-ELSD 3300 evaporative light scattering detector: Otai Technology, USA) coupled with a Grace Prevail Carbohydrate ES column (250 × 4.6 mm i.d., 5 mm) according to the previously reported method (17). The mobile phase consisted of 25% (v/v) H_2_O and 75% (v/v) acetonitrile. The solvent flow rate was 1.0 mL/min and the injection volume was 10 μL. The column temperature was maintained at 30°C. The drift tube temperature of the ELSD detector was 80°C, the flow rate of nitrogen gas was 1.5 L/min. The content of each sugar was expressed as mg/L.

### Determination of Free Amino Acids Contents

The apple juice collected at different time intervals was filtered through 0.45 μm membranes. The amino acids were analyzed by a L-8900 automatic amino acid analyzer (Hitachi Technology Company, Japan). The content of each free amino acid was expressed as mg/L.

### Determination of the Contents of Total Phenolics and Individual Phenolic Compounds

The method for the determination of total phenols was the Folin-Ciocalteu method (18). Specifically, 0.2 mL of diluted apple juice sample was mixed with 1.5 mL of 7.5% (w/v) Na_2_CO_3_ and 1.5 mL of Folin-Ciocalteu (10-fold dilution). After standing at 25°C in darkness for 40 min, and the absorbance was measured at 765 nm. The total phenolic content was standardized against gallic acid and expressed as gallic acid equivalents/L.

The phenolic substances were analyzed using a Shimadzu-HPLC system coupled with an Inertsil ODS-3 column (250 × 4.6 mm i.d., 5 mm) (LC-2010A, Shimadzu Corporation, Japan) according to the previously reported method (19, 20). The column was maintained at 25°C, and the injection volume was 20 μL. The binary mobile phase consisted of (A) glacial acetic acid aqueous (1%, v/v) and (B) glacial acetic acid methanol (1%, v/v). The samples were eluted at a flow rate of 0.6 mL/min with the following gradients: 0–10 min, 10–26% B; 10–25 min, 10–26% B; 25–45 min, 40–65% B; 45–55 min, 65–95% B; 55–58 min, 95–100% B; 58–65 min, 100% B. Flavonoids and phenolic acids in samples were detected at 350 and 280 nm respectively. The results were expressed in mg/L.

### Determination of Antioxidant Activities *in vitro*

The free radical scavenging activity of apple juice was assessed by the ABTS^·+^ method, as described in previous paper (21). The calibration curve was made using Trolox as the standard, and the results were expressed as mmol Trolox/L. Ferric reducing antioxidant power (FRAP) was determined according to our previously reported method (21). FeSO_4_ was employed as the standard to obtain the calibration curve, and the results were expressed as mmol Fe ^2+^/L.

### Statistical Analysis

The experiments were repeated three times. The test data were analyzed by Excel, Origin 8.0, Minitab, and SPSS 20.0 software. Significance was defined at *p* < 0.05, and Duncan's test was used.

## Results and Discussion

### Bacterial Growth and Change in pH During LAB Fermentation and Storage

**Figure 1:**
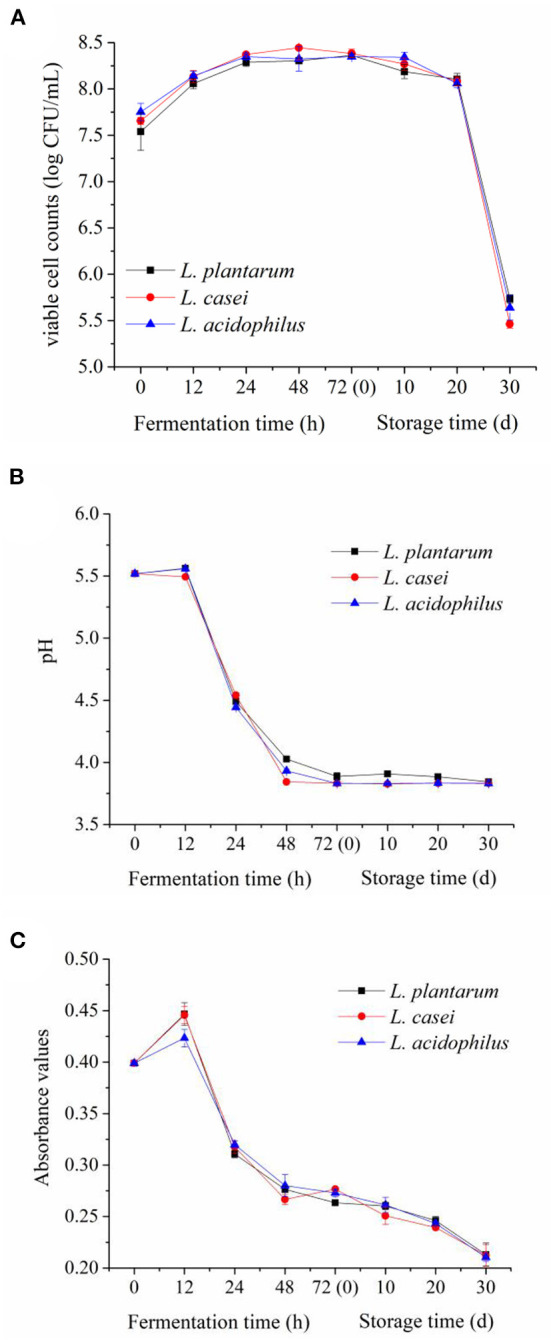
Evolutions of viable cell counts **(A)**, pH value **(B)** and browning index **(C)** in apple juices fermented by the three LAB strains during fermentation and refrigerated storage (4°C, 30 days).

Changes in viable cell counts and pH value of apple juice fermented with *L. acidophilus, L. casei* and *L. plantarum* during 72-h fermentation and the subsequent 30-day storage are shown in Figure 1, which can reflect the growth status of these strains. As can be seen from Figure 1A, *L. acidophilus, L. casei* and *L. plantarum* all grew well in the apple juice, and there was no significant difference in their viable cell count during the fermentation process (*p* ≥ 0.05). At the latter period of fermentation (from 48 to 72 h), the viable cell counts of *L. acidophilus, L. casei* and *L. plantarum* were still above 8.3 log CFU/mL. This result demonstrates that the growth of *L. acidophilus, L. casei* and *L. plantarum* metabolized vigorously. Meanwhile, this result also confirmed that apple juice can be a suitable substrate for the fermentation of the selected strains. Additionally, the viable cell counts of three LAB strains were maintained above 8.0 log CFU/mL in apple juice during the first 20 days of storage at 4°C. However, during the storage of 20–30 days, the viable cell counts of *L. casei* decreased rapidly to 5.4 ± 0.04 log CFU/mL, following *L. acidophilus* (5.6 ± 0.13 log CFU/mL) and *L. plantarum* (5.7 ± 0.04 log CFU/mL). These results are in line with the findings reported by Yoon et al. (22), which states that *L. casei* and *L. plantarum* were able to grow in cabbage juice without supplementing nutrients. With the increase of storage time, the substances that can be used by LAB in the apple juice were dramatically reduced with a marked accumulation of the metabolites (13). Eventually, the growth of the LAB reached the death phase, when the viable cell counts of LAB rapidly decreased.

LAB strains can metabolize sugar and produce organic acids, thus changing the pH value of apple juice during fermentation and storage (23). The change in pH values of apple juice fermented with *L. acidophilus, L. casei* and *L. plantarum* is shown in Figure 1B. The pH values of apple juice fermented by the three LAB strains had a minor change in the first 12 h of fermentation, and then experienced a rapid drop between 12 and 72 h (from 5.5~4.0). To be specific, after 48-h fermentation, the maximum reduction of pH value was obtained in apple juice fermented with *L. casei* (3.8 ± 0.02), following that fermented with *L. acidophilus* (3.9 ± 0.01) and *L. plantarum* (4.0 ± 0.02). In the storage period, the pH value remained stable, and there was no significant difference among the juices fermented by the three different strains (*p* ≥ 0.05). The variations of the pH values were consistent with the report of Chen et al. (10), who studied the effects of four LAB strains on the flavor characteristics of fermented apple juice.

### Change in Browning Index During LAB Fermentation and Storage

Color is an important quality indicator for apple juice, and the fermentation of LAB in fruit and vegetable substrates can change their color (11). The change in color of apple juice fermented with *L. acidophilus, L. casei* and *L. plantarum* during the 72-h fermentation and the subsequent 30-day storage are shown in Figure 1C. The Browning Index value of apple juice increased slightly during the first 12 h of fermentation, and then decreased rapidly during the fermentation between 12 and 24 h, which was consistent with the variation of pH. The Browning Index value showed a downward trend during the period of 24-h fermentation and 30-day storage at 4°C. This may be due to the metabolism of phenolics in apple juice caused by LAB fermentation and the decrease of pH in apple juice, which inhibited the color change caused by Maillard reaction (24).

### Changes in Organic Acids and Sugars Contents During LAB Fermentation and Storage

The changes in organic aicds and sugars in apple juice fermented with *L. acidophilus, L. casei* and *L. plantarum* during the 72-h fermentation and the subsequent 30-day storage are summarized in Supplementary Table 1. Organic acids are important products derived from LAB metabolism, which can improve the flavor and palatability of fruit juices (25). Meanwhile, organic acids can increase the acidity of the juice and inhibit the growth of spoilage and pathogen microorganisms. Therefore, organic acids have a profound influence on the storage characteristics and flavor of fermented fruit and vegetable juices (25). As shown in Supplementary Table 1, six organic acids including oxalic acid, pyruvic acid, malic acid, lactic acid, citric acid, and succinic acid can be identified in the fermented apple juice with *L. acidophilus, L. casei* and *L. plantarum*.

Lactic acid content was increased significantly after fermentation owing to the biosynthesis pathway of LAB metabolism, and the highest concentration was 4,191.6 ± 31.6 mg/L by *L. case*i. Malic acid is the main source of sour flavor in unfermented apple juice. Before fermentation, it had the highest content of 1,166.5 ± 14.7 mg/L in apple juice (26, 27). Malic acid can be bio-transferred to lactic acid under the catalysis of the malolactic enzyme produced by LAB, thus weakening the acidity and providing a mildly sour taste of apple juice (28). The existence of this metabolic pathway can be confirmed by the similarity between the increasing amount of lactic acid content and the decreasing amount of malic acid content.

Citric acid content (730.7 ± 13.1 mg/L in unfermented juice) was decreased after lactic acid fermentation as a result of its decomposition into various products, such as diacetyl, lactic acid, and acetic acid (29). Pyruvate is an intermediate product of the basic metabolic pathways of organisms such as tricarboxylic acid cycle, glycolysis, and other metabolic pathways. The pyruvic acid content experienced an increasing trend and reached a maximum concentration of 130.2 ± 1.4 mg/L after fermentation by *L. plantarum* for 24 h, and then followed a decreasing trend. It has been confirmed that pyruvic acid can be produced from glucose through glycolysis, which may be related to the rising phase of pyruvic acid. In addition, pyruvic acid can also be used to form the amino acid alanine and converted into lactic acid or ethanol via fermentation, which may be associated with its decline at the end of fermentation (30). The trends in the content of oxalic acid and succinic acid in fermented apple juice were similar to that of pyruvic acid. In addition, the content of total organic acids increased during fermentation and then remained stable during the storage period, which may be due to the inhibition of the metabolism of lactic acid bacteria at low temperatures.

Before fermentation, fructose is the major sugar (52.0 ± 2.3 mg/L) in apple juice. During the fermentation process, the content of glucose in all fermented samples experienced a continuous decrease from 17.7 ± 0.8 mg/L to about 11.7 ± 0.1 mg/L, although there was no significant difference in the content of glucose among the apple juice fermented with different microbial strains (*p* ≥ 0.05). This trend can be explained by the reason that the fermentation of the selected LAB strains is facultative heterologous fermentation, and glucose and fructose are converted into lactic acid through the Embden-Meyerhof pathway. The content of sucrose in apple juice fermented with *L. acidophilus, L. casei* and *L. plantarum* was reduced continuously, which can be attributed to the hydrolysis of sucrose by galactosidase in LAB strains. This trend is consistent with the results of Wang et al. (31) who used LAB strains to ferment soy milk. The contents of glucose, fructose and sorbitol were also increased significantly (*P* < 0.05) during storage. This may be due to the inhibition of the respiration of LAB strains by monosaccharides in an environment of low temperature and high acidity. Meanwhile, the degradation of sucrose by galactosidase may also take place in LAB strains (32–34).

### Change of Free Amino Acids During LAB Fermentation and Storage

**Figure 2:**
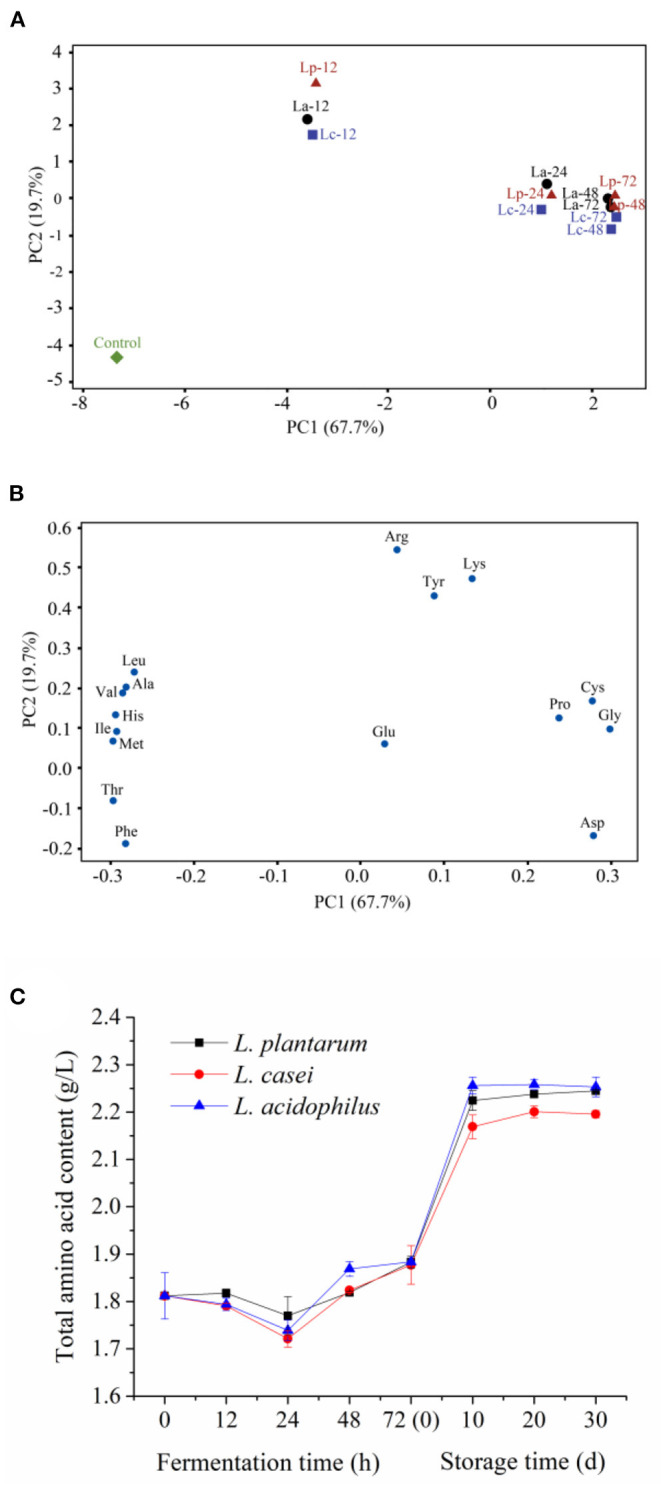
Principal component analysis of free amino acid profile in apple juice during fermentation by the three LAB strains [**(A)**, score plot; **(B)**, loading plot] and changes in total free amino acids **(C)**. “Control,” Unfermented apple juice; “La,” Apple juice fermented by *L. acidophilus*; “Lp”, Apple juice fermented by *L. plantarum*; “Lc”, Apple juice fermented by *L. casei*. In **(A)**, the number followed with the name of strain indicated the time of fermentation.

Amino acids are important flavoring compounds and their compositions have a profound influence on the evaluation of sensory properties of apple juice (35). For LAB fermentation, amino acids may first undergo decarboxylation, deamination, transamination, and desulfurization reactions, followed by amine conversion. Eventually, the aldehydes generated from amino acid metabolism are oxidized or reduced to the corresponding carboxylic acids and alcohols (36). Seventeen free amino acids could be detected in the apple juice samples fermented by the three LAB strains. Among them, Thr possessed the highest amount in unfermented samples (695.3 ± 13.2 mg/L). To clarify the effect of LAB strains on the metabolism of amino acids, two-dimensional analysis of the principal component analysis (PCA) was used to analyze the profile of free amino acids during 72-h fermentation. Meanwhile, the original data about the content of each amino acid are prepared in Table 1. Two principal components, PC1 and PC2, were extracted that accounted for 67.7% and 19.7% of the total variance in seventeen variable systems, respectively. The unfermented sample was distributed on the negative side of PC1. During 72-h fermentation, a significant shift of the distribution could be observed among fermented apple juice (Figure 2A). This phenomenon indicates that the profile of amino acids in apple juice changed obviously during 72-h fermentation. For example, the PC1 value of unfermented apple juice was−7.31. After fermentation for 72 h, the PC1 values for apple juice fermented with *L. acidophilus, L. plantarum*, and *L. casei* increased to 2.42, 2.49, and 2.47. According to the loading plot (Figure 2B), Leu, Ala, Val, His, Ile, Met, Thr and Phe distributed on the negative side of PC1, and Tyr, Lys, Pro, Arg, Glu, Asp, Cys and Gly distributed on the positive side. These amino acids placed to the right such as Pro, Cys and Gly in the loading plot were close and positively correlated. However, amino acids on the negative side were negatively correlated. In addition, some amino acids including Leu, Val, Ala, His, Ile, Met, Thr, Phe, Pro, Cys, Gly and Asp distributed far from the origin of the first PC. Therefore, LAB fermentation of apple juice may have a higher influence on the content of these amino acids. Based on the locations of samples and the attributes, it can be found that the contents of Leu, Ala, Val, His, Ile, Met, Thr and Phe in apple juice were generally decreased with fermentation. In contrast, the lactic acid fermentation led to the increment of the amounts of Tyr, Lys, Pro, Arg, Glu, Asp, Cys and Gly. Besides, it can be observed that the fermented apple juice by the three microbial strains at the same stage had similar PC1 values, indicating that PC2 is mainly responsible for the discrimination of the samples fermented by different strains. For example, after 72-h fermentation, the contents of Asp, Cys and Gly in apple juice fermented with *L. acidophilus, L. plantarum*, and *L. casei* were 979.4 ± 1.0 mg/L, 972.9 ± 1.0 mg/L, 969.3 ± 16.8 mg/L; 6.2 ± 0 mg/L, 6.2 ± 0.2 mg/L, 5.8 ± 0.1 mg/L and 80.3 ± 1.2 mg/L, 86.9 ± 1.7 mg/L, 85.3 ± 1.2 mg/L, respectively. And the PC2 values of Asp, Cys and Gly were−0.17, 0.17, and 0.10 (Figure 2B). This result implies that different strains have different capacities to metabolize some amino acids such as Asp, Cys and Gly and others.

**Table 1:** Changes in the contents of free amino acids (mg/L) of apple juice during lactic acid fermentation and the subsequent refrigerated storage period (4°C, 30 days).

**Amino**	**Strains**	**Time**							
**acid**									
		**Fermentation for 0 h**	**Fermentation for 12 h**	**Fermentation for 24 h**	**Fermentation for 48 h**	**Fermentation for 72 h (Storage for 0 d)**	**Storage for 10 d**	**Storage for 20 d**	**Storage for 30 d**
Asp	*L. acidophilus*	678.8 ± 31.8^Ad^	662.3 ± 1.9^Bd^	834.4 ± 15.7^Ac^	974.1 ± 14.2^Ab^	979.4 ± 1.0^Ab^	1,529.7 ± 12.5^Aa^	1522.8 ± 1.6^Aa^	1512 ± 4.5^Aa^
	*L. casei*	678.8 ± 31.8^Ad^	672.4 ± 7.3^ABd^	835.3 ± 17^Ac^	933.4 ± 5.7^Bb^	969.3 ± 16.8^Ab^	1,443 ± 20.9^Ba^	1460.3 ± 6.2^Ba^	1,458.2 ± 2.5^Ca^
	*L. plantarum*	678.8 ± 31.8^Ad^	676.6 ± 1.5^Ad^	852.9 ± 33.6^Ac^	926.7 ± 7.2^Bb^	972.9 ± 1.0^Ab^	1,496.4 ± 20.2^ABa^	1492.7 ± 17.4^ABa^	1,489.3 ± 9.8^Ba^
Thr	*L. acidophilus*	695.3 ± 13.2^Aa^	522.7 ± 2.7^Ab^	404.3 ± 6.2^Ac^	385.5 ± 1.8^Ad^	373.9 ± 2.6^Ad^	ND	ND	ND
	*L. casei*	695.3 ± 13.2^Aa^	521.5 ± 4.6^Ab^	400.2 ± 0.3^Ac^	379.4 ± 2.3^Ad^	371.4 ± 2.9^Ad^	ND	ND	ND
	*L. plantarum*	695.3 ± 13.2^Aa^	533.6 ± 8.2^Ab^	412.9 ± 8.6^Ac^	378.2 ± 5.3^Ad^	370.6 ± 6.8^Ad^	ND	ND	ND
Ser	*L. acidophilus*	ND	ND	ND	ND	ND	ND	ND	ND
	*L. casei*	ND	ND	ND	ND	ND	ND	ND	ND
	*L. plantarum*	ND	ND	ND	ND	ND	ND	ND	ND
Glu	*L. acidophilus*	200.9 ± 3.2^Ad^	213.4 ± 2.5^Ac^	184.6 ± 0.8^Ae^	205.3 ± 1.5^Bd^	218.4 ± 1.6^Bb^	292.2 ± 3.1^Aa^	294.7 ± 1.3^Ba^	294.1 ± 0.4^Ba^
	*L. casei*	200.9 ± 3.2^Ad^	211.2 ± 1.1^Ac^	185 ± 2.3^Ae^	212 ± 0.6^Ac^	227.7 ± 1.7^Ab^	293.7 ± 6.1^Aa^	300.5 ± 2.2^Aa^	299.9 ± 2.4^Aa^
	*L. plantarum*	200.9 ± 3.2^Ad^	210.1 ± 1.8^Ac^	189.6 ± 4.8^Ae^	206.1 ± 2.8^Bcd^	224.3 ± 0.5^Ab^	298.9 ± 1.1^Aa^	300.1 ± 0.5^Aa^	301.1 ± 1.1^Aa^
Gly	*L. acidophilus*	4 ± 0.1^Af^	47 ± 0^Be^	72.5 ± 0.9^Bd^	77.8 ± 1.6^Bc^	80.3 ± 1.2^Bb^	100.2 ± 0.6^Ba^	100.1 ± 1.3^Ba^	101.3 ± 1.2^Ba^
	*L. casei*	4 ± 0.1^Ae^	46.9 ± 0.2^Bd^	76.8 ± 1.8^ABc^	83 ± 1.4^Ab^	85.3 ± 1.2^Ab^	104.9 ± 2^ABa^	106.2 ± 1.9^Aa^	106.5 ± 1.2^Aa^
	*L. plantarum*	4 ± 0.1^Ae^	48.2 ± 0.1^Ad^	77.6 ± 1.7^Ac^	84.8 ± 1.4^Ab^	86.9 ± 1.7^Ab^	106.8 ± 1.6^Aa^	107.7 ± 1.7^Aa^	108.9 ± 1.8^Aa^
Ala	*L. acidophilus*	33.3 ± 0.1^Ab^	37.8 ± 0.9^Aa^	8.3 ± 0.8^Ac^	1.9 ± 0.2^Ad^	1.9 ± 0^Ad^	ND	ND	ND
	*L. casei*	33.3 ± 0.1^Ab^	36.8 ± 0.9^Aa^	7.7 ± 0.4^Ac^	1.9 ± 0^Ad^	1.9 ± 0.2^Ad^	ND	ND	ND
	*L. plantarum*	33.3 ± 0.1^Ab^	39 ± 1.1^Aa^	9.9 ± 1^Ac^	2 ± 0.2^Ad^	1.9 ± 0.1^Ad^	ND	ND	ND
Cys	*L. acidophilus*	3.1 ± 0.1^Ad^	5.1 ± 0^Ac^	6.5 ± 0.3^Aab^	6.5 ± 0^Aa^	6.2 ± 0^Ab^	ND	ND	ND
	*L. casei*	3.1 ± 0.1^Ad^	5 ± 0^Ac^	6 ± 0.1^Aa^	5.9 ± 0^Bab^	5.8 ± 0.1^Bb^	ND	ND	ND
	*L. plantarum*	3.1 ± 0.1^Ac^	5.2 ± 0^Ab^	6.4 ± 0.4^Aa^	6.4 ± 0.2^Aa^	6.2 ± 0.2^Aa^	ND	ND	ND
Val	*L. acidophilus*	31.5 ± 0.1^Ab^	35.3 ± 0.3^Aa^	8.7 ± 0.9^Ac^	2.1 ± 0.2^Ad^	2.7 ± 0.2^Ad^	8.4 ± 0.4^Ac^	6.9 ± 3.5^Ac^	8.2 ± 0.7^Ac^
	*L. casei*	31.5 ± 0.1^Ab^	34.2 ± 0.4^Aa^	6 ± 0.3^Bd^	1.5 ± 0.1^Be^	2.2 ± 0.2^Ae^	7.5 ± 0.4^Ac^	8 ± 0.4^Ac^	7.7 ± 0.7^Ac^
	*L. plantarum*	31.5 ± 0.1^Ab^	34.8 ± 0.5^Aa^	7.7 ± 0.8^ABc^	1.7 ± 0^Be^	2.3 ± 0.2^Ae^	4.1 ± 0.3^Bde^	6.3 ± 3.4^Acd^	8.3 ± 0.5^Ac^
Met	*L. acidophilus*	10.4 ± 0.5^Aa^	8 ± 0^Aab^	2.9 ± 1^Abc^	1.1 ± 0.5^Ac^	1.2 ± 0.3^ABc^	4.3 ± 1.1^Abc^	4.7 ± 1.3^Abc^	11.4 ± 6.4^Aa^
	*L. casei*	10.4 ± 0.5^Aab^	7.7 ± 0^Bb^	0.9 ± 0.5^Ac^	0.6 ± 0^Ac^	0.9 ± 0^Bc^	9.7 ± 3.8^Aab^	13 ± 1.1^Aa^	10.3 ± 5^Aab^
	*L. plantarum*	10.4 ± 0.5^Aab^	7.9 ± 0.2^ABabc^	1.3 ± 0^Ac^	1.1 ± 0.5^Ac^	1.5 ± 0^Ac^	3.5 ± 0.1^Abc^	9.6 ± 8.8^Aab^	13.8 ± 2.2^Aa^
Ile	*L. acidophilus*	59.6 ± 1.6^Aa^	49.1 ± 0.5^Ab^	7.4 ± 0.3^Ac^	1.4 ± 0.1^Ad^	1.8 ± 0.2^Ad^	2.4 ± 0.6^Ad^	3.9 ± 3.1^Acd^	4 ± 2.6^Acd^
	*L. casei*	59.6 ± 1.6^Aa^	46.9 ± 0.6^Ab^	3.9 ± 0.2^Bc^	0.7 ± 0.1^Bd^	1.2 ± 0.1^Ad^	0.9 ± 0.2^Bd^	1.6 ± 1^Ad^	1.8 ± 0.6^Ad^
	*L. plantarum*	59.6 ± 1.6^Aa^	48.4 ± 1.1^Ab^	5.9 ± 0.9^Ac^	0.9 ± 0.3^ABd^	1.5 ± 0.3^Ad^	1.3 ± 0.3^ABd^	3.4 ± 3.2^Acd^	3.4 ± 3.2^Acd^
Leu	*L. acidophilus*	16.7 ± 0.3^Ab^	22.6 ± 0.4^Aa^	4.1 ± 0.1^Acd^	1.8 ± 0.3^Ad^	2.6 ± 0.3^Ad^	3.9 ± 0.4^Acd^	7.1 ± 2.8^Ac^	6.5 ± 3.1^Ac^
	*L. casei*	16.7 ± 0.3^Ab^	21.8 ± 0.5^Aa^	3.1 ± 0.2^Bc^	0 ± 0^Bf^	1.2 ± 0.6^Ae^	2.2 ± 0.5^Ad^	2.2 ± 0.5^Ad^	2.4 ± 0.1^Acd^
	*L. plantarum*	16.7 ± 0.3^Ab^	22.4 ± 0.2^Aa^	3.9 ± 0.3^Acde^	1.4 ± 0.5^Ae^	2.1 ± 0.6^Ade^	3.4 ± 0.7^Acde^	5.8 ± 4.1^Acd^	6.7 ± 2.9^Ac^
Tyr	*L. acidophilus*	ND	3.2 ± 0.1^Aa^	3.5 ± 0.2^Aa^	3.9 ± 0.1^Aa^	4 ± 0^Aa^	ND	3.6 ± 0.6^Aa^	2 ± 2.8^Aab^
	*L. casei*	ND	3 ± 0^Ad^	3.7 ± 0.1^Ab^	3.4 ± 0.1^Bc^	4.1 ± 0.2^Aa^	ND	ND	ND
	*L. plantarum*	ND	7.9 ± 7^Aa^	3.3 ± 0^Aab^	3.9 ± 0.1^Aab^	3.9 ± 0^Aab^	ND	2 ± 2.9^Aab^	3.7 ± 0.4^Aab^
Phe	*L. acidophilus*	12.9 ± 0.1^Aa^	8.9 ± 0.3^Ac^	7.1 ± 0^Ad^	7.1 ± 0.1^Ad^	7.2 ± 0.1^Ad^	12 ± 0.3^Ab^	12.1 ± 0.1^Ab^	11.8 ± 0^Bb^
	*L. casei*	12.9 ± 0.1^Aa^	8.9 ± 0.3^Ac^	7.2 ± 0.1^Ad^	6.8 ± 0.1^Be^	7.1 ± 0.2^Ade^	11.7 ± 0.1^Ab^	11.8 ± 0.1^ABb^	11.9 ± 0^Ab^
	*L. plantarum*	12.9 ± 0.1^Aa^	9 ± 0.4^Ac^	7.2 ± 0^Ad^	7 ± 0^Ad^	7.2 ± 0.1^Ad^	11.7 ± 0.1^Ab^	11.7 ± 0.1^Bb^	11.9 ± 0.1^ABb^
Lys	*L. acidophilus*	25.4 ± 0.1^Ad^	51 ± 0.7^Ab^	51.8 ± 0.6^Ab^	46.9 ± 0.1^ABc^	46 ± 0.1^Ac^	60.9 ± 0.8^Aa^	61.1 ± 0.5^Aa^	60.6 ± 0.2^Aa^
	*L. casei*	25.4 ± 0.1^Ad^	49.9 ± 0.7^Ab^	49.6 ± 0.1^Ab^	44.8 ± 0.4^Bc^	44 ± 0.5^Ac^	57.8 ± 0.6^Aa^	58.2 ± 1^Aa^	58 ± 0.1^Aa^
	*L. plantarum*	25.4 ± 0.1^Ac^	50.1 ± 0.5^Ab^	49.4 ± 3.9^Ab^	47.4 ± 1.3^Ab^	46.5 ± 1.4^Ab^	60.9 ± 1.7^Aa^	61.1 ± 1.5^Aa^	60.7 ± 1.9^Aa^
His	*L. acidophilus*	26 ± 0.1^Aa^	25.5 ± 0.2^Ab^	21.1 ± 0.1^Ad^	19.4 ± 0^Ae^	19.5 ± 0^Ae^	24 ± 0.3^Ac^	24.1 ± 0.1^Ac^	24 ± 0.1^Ac^
	*L. casei*	26 ± 0.1^Aa^	25 ± 0.2^Ab^	20.5 ± 0.2^Ad^	18.8 ± 0.1^Be^	19 ± 0.1^Be^	23 ± 0.2^Bc^	23.3 ± 0.4^Bc^	23.3 ± 0.2^Bc^
	*L. plantarum*	26 ± 0.1^Aa^	25.4 ± 0.2^Ab^	20.8 ± 0.5^Ad^	19.3 ± 0.1^Ae^	19.4 ± 0.2^ABe^	23.6 ± 0.2^ABc^	23.6 ± 0.1^ABc^	23.6 ± 0.1^ABc^
Arg	*L. acidophilus*	10.8 ± 0.1^Ae^	45.3 ± 0.7^Aa^	36.3 ± 0.4^Ac^	33.5 ± 0.3^Ad^	34.2 ± 0.5^Ad^	40.7 ± 0.8^Ab^	41.6 ± 0.5^Ab^	41.2 ± 0.2^Ab^
	*L. casei*	10.8 ± 0.1^Ae^	44.4 ± 0.7^Aa^	34.9 ± 0.2^Ac^	32.3 ± 0.5^Ad^	32.9 ± 0.4^Ad^	38.9 ± 0.6^Ab^	39.5 ± 1^Ab^	39.5 ± 0.4^Bb^
	*L. plantarum*	10.8 ± 0.1^Ae^	44.4 ± 0.6^Aa^	35.4 ± 1^Ac^	32.9 ± 0.4^Ad^	33.3 ± 0.7^Ad^	39.9 ± 0.5^Ab^	40.1 ± 0.5^Ab^	39.8 ± 0.8^ABb^
Pro	*L. acidophilus*	ND	6.3 ± 0.6^Ac^	6.7 ± 0.1^Ac^	9.9 ± 0.6^Ab^	11.1 ± 0.6^Ab^	53.7 ± 0.1^Aa^	54.8 ± 2.8^Aa^	55.1 ± 0.4^Aa^
	*L. casei*	ND	5.8 ± 0.3^Acd^	2.6 ± 3.7^Ade^	9 ± 0.2^Abc^	10.7 ± 0.5^Ab^	55.5 ± 2.2^Aa^	56 ± 0.4^Aa^	56 ± 0.3^Aa^
	*L. plantarum*	ND	5.8 ± 0.7^Ac^	6.9 ± 0.3^Ac^	8.9 ± 0.9^Ab^	10.4 ± 0.9^Ab^	53.5 ± 1.7^Aa^	53.9 ± 0.7^Aa^	53.4 ± 0.4^Ba^

*Results are expressed as mean ± standard deviation from the three replicates. ND refers to not detected. Values with different letters indicated a significant difference (p < 0.05)*.

The contents of Asp and Gly were increased significantly during fermentation (*P* < 0.05), which may improve the flavor of fermented apple juice (37). LAB can metabolize threonine to pyruvate and ammonia through threonine dehydratase, resulting in a significant decrease in threonine content (*P* < 0.05) during the first 24-h fermentation. It has been reported that lactic acid bacteria can alleviate environmental acid stress by increasing the expression of enzymes consuming cytoplasm (38). Ammonia produced by amino acid metabolism can accept protons and promote acid-base balance in microbial cells (29), which is confirmed by the increase in ammonia contents from 3.5 ± 0.1 mg/L to 121.0 ± 0.2 mg/L, 121.0 ± 2.4 mg/L, 120.2 ± 0.01 mg/L during fermented by *L. acidophilus, L. plantarum*, and *L. casei*, respectively. Moreover, the contents of Phe, Val, Ile and Met also showed a downward trend throughout fermentation, which may be related to the decarboxylase (4) and transaminase (39, 40) produced by LAB. Decarboxylase decomposes free amino acids to produce primary amine and release carbon dioxide, while transaminases can catalyze the inter-conversion of amino acids into alpha-ketonic acid (38) and the transamination reaction between branched-chain amino acids (i.e., leucine) and alpha-keto-beta-methyl valerate/alpha-keto isopropionate (41).

During storage, almost all the mentioned amino acids were increased significantly during the first 10-day storage (*P* < 0.05). To be specific, the contents of Asp, Phe and Gly in apple juice fermented by *L. plantarum* increased from about 972.9 ± 1.0 mg/L, 7.2 ± 0.1 mg/L and 86.9 ± 1.7 mg/L to approximately 1496.4 ± 20.2 mg/L, 11.7 ± 0.1 mg/L and 106.8 ± 1.6 mg/L, respectively. Meanwhile, the contents of Met, Ile, Leu had no obvious changes in the early period of storage. In the contrast, the content of Thr was decreased from 370.6 mg/L to below 1 mg/L. In the subsequent storage period, there was no significant change in the content of all the amino acids (*P* ≥ 0.05).

Besides, the content of total amino acids in apple juice fermented by three LAB strains was studied. The results are shown in Figure 2C. The total amino acid content in fermented apple juice was decreased slightly in the first 24 h of fermentation, and then increased gradually. During the first 10 days of storage at 4°C, the content of total amino acids in apple juice fermented by *L. acidophilus, L. plantarum*, and *L. casei* were increased from 1.88 ± 0.002 g/L, 1.88 ± 0.01, and 1.88 ± 0.04 g/L to 2.26 ± 0.02 g/L, 2.22 ± 0.02 g/L, and 2.27 ± 0.03 g/L, respectively. Then maintained at this level until the end of storage.

### Evolution of Phenolic Profile During LAB Fermentation and Storage

Gallic acid, protocatechuic acid, catechin, proanthocyanidin B_2_, chlorogenic acid, *p*-hydroxybenzoic acid, coffee acid, syringic acid, phloxic acid, *p*-coumaric acid, ferulic acid, cinnamaldehyde, *p*-vinyl guaiacol, rutin and quercitrin can be tentatively identified in the fermented apple juice. Gallic acid, protocatechuic acid, catechin, proanthocyanidin B_2_, caffeic acid, rutin ferulate and quercetin were the main phenolic substances in fermented apple juice, and their contents were summarized in Table 2. After 48-h fermentation by *L. acidophilus, L. plantarum*, and *L. casei*, the content of ferulic acid was declined by 11.1, 18.5, and 11.1%, and the caffeic acid content was reduced by 41.7, 50, and 50%, respectively. These results indicated that the selected LAB strains were able to biotransform ferulic acid and caffeic acid. This phenomenon can be explained by two possible pathways. One is that phenolic acid decarboxylases synthesized by LAB can catalyze the non-oxidative decarboxylation of phenolic acids to generate their corresponding *p*-vinyl derivatives (42). Another reason is that ferulic acid and caffeic acid could be transformed to dihydrocaffeic acid and dihydroferulic acids through the side chain hydrogenation (43–46). The ferulic acid content in apple juice fermented by *L. plantarum* was decreased faster than those fermented by *L. acidophilus* and *L. casei* (*P* < 0.05), guessing that *L. plantarum* was more capable to synthesize the enzymes involved in the metabolism of ferulic acid.

**Table 2:** Changes in the contents of individual phenolic compounds (mg/L) of apple juices during lactic acid fermentation and the subsequent refrigerated storage period (4°C, 30 days).

**organic**	**strains**	**Time**							
		**Fermentation for 0 h**	**Fermentation for 12 h**	**Fermentation for 24 h**	**Fermentation for 48 h**	**Fermentation for 72 h (Storage for 0 d)**	**Storage for 10 d**	**Storage for 20 d**	**Storage for 30 d**
Gallic acid	*L. acidophilus*	15.4 ± 0.06^ef^	12.8 ± 0.54^de^	16.8 ± 0.11^f^	19.8 ± 0.10^gh^	21.3 ± 0.23^h^	9.9 ± 2.23^c^	4.5 ± 0.45^a^	3.2 ± 0.13^a^
	*L. casei*	15.4 ± 0.06^ef^	14.3 ± 1.94^ef^	16.5 ± 0.11^f^	19.6 ± 0.05^gh^	21.6 ± 0.16^h^	10.5 ± 2.57^cd^	4.3 ± 0.63^a^	2.8 ± 0.42^a^
	*L. plantarum*	15.4 ± 0.06^ef^	12.2 ± 0.37^d^	16.5 ± 0.32^f^	18.9 ± 0.14^g^	20.8 ± 0.14^gh^	7.5 ± 1.57^b^	3.2 ± 0.52^a^	2.9 ± 0.15^a^
Protocatechuic acid	*L. acidophilus*	17.1 ± 0.50^ab^	16.0 ± 1.45^c^	24.9 ± 0.40^c^	23.9 ± 0.87^c^	24.4 ± 0.09^c^	16.2 ± 0.84^ab^	18.8 ± 1.19^b^	17.4 ± 1.02^ab^
	*L. casei*	17.1 ± 0.50^ab^	20.1 ± 1.70^bc^	23.0 ± 2.31^c^	24.4 ± 0.73^c^	25.1 ± 0.31^c^	18.2 ± 0.60^ab^	19.9 ± 1.23^bc^	17.9 ± 0.68^ab^
	*L. plantarum*	17.1 ± 0.50^ab^	17.4 ± 0.95^ab^	20.0 ± 0.97^bc^	22.5 ± 2.69^c^	22.8 ± 2.57^c^	18.9 ± 2.50^b^	19.4 ± 0.37^b^	18.1 ± 1.24^ab^
Catechin	*L. acidophilus*	8.9 ± 0.08^a^	8.6 ± 1.73^a^	10.3 ± 0.16^c^	9.6 ± 0.35^bc^	10.1 ± 0.14^bc^	12.6 ± 0.89^d^	13.5 ± 0.65^de^	12.6 ± 0.68^d^
	*L. casei*	8.9 ± 0.08^a^	9.7 ± 0.27^bc^	9.9 ± 0.14^bc^	9.7 ± 0.36^bc^	10.5 ± 0.37^c^	14.7 ± 0.29^e^	13.5 ± 0.18^de^	12.9 ± 0.35^de^
	*L. plantarum*	8.9 ± 0.08^a^	7.7 ± 0.18^a^	9.6 ± 1.17^bc^	9.0 ± 0.77^b^	9.9 ± 0.27^bc^	14.0 ± 0.48^e^	13.7 ± 0.17^de^	12.8 ± 0.63^d^
Procyanidin B2	*L. acidophilus*	78.7 ± 6.78^f^	43.1 ± 8.73^c^	58.9 ± 1.08^e^	56.5 ± 2.40^de^	57.4 ± 0.38^de^	28.4 ± 1.77	27.7 ± 1.58^ab^	24.9 ± 1.59^ab^
	*L. casei*	78.7 ± 6.78^f^	52.3 ± 0.73^d^	57.4 ± 1.43^de^	57.6 ± 1.65^de^	59.4 ± 0.49^e^	31.9 ± 1.47^b^	28.1 ± 0.73^ab^	24.7 ± 1.34^ab^
	*L. plantarum*	78.7 ± 6.78^f^	42.7 ± 0.99^c^	56.9 ± 3.48^de^	55.8 ± 2.29^de^	57.8 ± 0.60^de^	30.0 ± 0.88^b^	28.1 ± 0.54^ab^	23.9 ± 2.67^a^
Chlorogenic acid	*L. acidophilus*	116.0 ± 0.26^g^	97.8 ± 2.66^c^	111.0 ± 0.43^e^	107.8 ± 1.25^de^	106.8 ± 0.32^de^	99.0 ± 2.90^ab^	98.7 ± 1.76^ab^	96.3 ± 0.89^ab^
	*L. casei*	116.0 ± 0.26^g^	99.7 ± 0.53^d^	110.5 ± 0.57^de^	108.7 ± 1.10^de^	107.9 ± 0.31^e^	105.2 ± 2.65^b^	97.9 ± 0.36^ab^	95.8 ± 0.79^ab^
	*L. plantarum*	116.0 ± 0.26^g^	94.2 ± 0.76^c^	110.0 ± 2.08^de^	107.4 ± 1.48^de^	106.9 ± 0.52^de^	100.9 ± 1.59^b^	98.2 ± 0.49^ab^	96.1 ± 1.09^a^
P-hydroxybenzoic acid	*L. acidophilus*	19.3 ± 0.18^d^	15.0 ± 1.64^a^	18.2 ± 0.15^cd^	20.1 ± 2.36^de^	21.4 ± 0.14^e^	15.2 ± 0.82^a^	15.8 ± 0.42^bc^	15.2 ± 0.50^a^
	*L. casei*	19.3 ± 0.18^d^	15.9 ± 0.17^bc^	17.8 ± 0.22^c^	21.6 ± 0.50^e^	21.5 ± 0.19^e^	16.9 ± 0.43^c^	15.7 ± 0.17^bc^	15.3 ± 0.27^b^
	*L. plantarum*	19.3 ± 0.18^d^	13.7 ± 0.14^a^	17.4 ± 0.96^c^	20.6 ± 1.20^de^	21.2 ± 0.10^e^	16.3 ± 0.43^bc^	15.9 ± 0.12^bc^	15.4 ± 0.42^bc^
Caffeic acid	*L. acidophilus*	1.2 ± 0.27^b^	1.0 ± 0.81^b^	1.2 ± 0.09^b^	0.7 ± 0.42^ab^	1.6 ± 0.07^bc^	2.0 ± 0.50^c^	3.0 ± 0.22^d^	3.0 ± 0.42^d^
	*L. casei*	1.2 ± 0.27^b^	1.6 ± 0.15^bc^	1.0 ± 0.11^b^	0.6 ± 0.29^ab^	2.0 ± 0.09^c^	3.0 ± 0.13^d^	3.1 ± 0.08^d^	3.2 ± 0.26^d^
	*L. plantarum*	1.2 ± 0.27^b^	0.0 ± 0.05^a^	0.4 ± 0.58^ab^	0.6 ± 0.42^ab^	1.9 ± 0.04^c^	3.0 ± 0.19^d^	3.3 ± 0.12^d^	3.0 ± 0.34^d^
Ferulic acid	*L. acidophilus*	5.4 ± 0.08^e^	5.1 ± 0.93^d^	5.0 ± 0.05^d^	4.8 ± 0.05^cd^	4.6 ± 0.19^cd^	0.4 ± 0.19^a^	0.8 ± 0.10^a^	0.7 ± 0.18^a^
	*L. casei*	5.4 ± 0.08^e^	5.4 ± 0.32^d^	4.9 ± 0.02^cd^	4.8 ± 0.06^cd^	4.8 ± 0.04^cd^	0.9 ± 0.02^a^	0.9 ± 0.03^a^	0.8 ± 0.11^a^
	*L. plantarum*	5.4 ± 0.08^e^	3.7 ± 0.00^b^	4.4 ± 0.56^c^	4.4 ± 0.53^c^	4.7 ± 0.04^cd^	0.8 ± 0.12^a^	0.9 ± 0.03^a^	0.8 ± 0.03^a^
Rutin	*L. acidophilus*	2.3 ± 0.00^bc^	2.2 ± 0.05^b^	2.5 ± 0.27^c^	2.4 ± 0.19^c^	2.6 ± 0.24^c^	0.3 ± 0.02^a^	0.3 ± 0.05^a^	0.3 ± 0.02^a^
	*L. casei*	2.3 ± 0.00^bc^	2.3 ± 0.08^bc^	2.2 ± 0.01^bc^	2.3 ± 0.02^bc^	2.3 ± 0.01^bc^	0.3 ± 0.02^a^	0.3 ± 0.02^a^	0.3 ± 0.03^a^
	*L. plantarum*	2.3 ± 0.00^bc^	2.2 ± 0.02^bc^	2.2 ± 0.01^bc^	2.3 ± 0.02^bc^	2.3 ± 0.00^bc^	0.4 ± 0.19^a^	0.3 ± 0.00^a^	0.3 ± 0.20^a^
Quercetin	*L. acidophilus*	2.1 ± 0.00^bc^	2.1 ± 0.12^bc^	2.2 ± 0.21^c^	2.2 ± 0.18^bc^	2.2 ± 0.19^c^	0.2 ± 0.01^a^	0.2 ± 0.03^a^	0.2 ± 0.02^a^
	*L. casei*	2.1 ± 0.00^bc^	2.0 ± 0.01^b^	2.1 ± 0.01^bc^	2.1 ± 0.01^bc^	2.1 ± 0.01^bc^	0.2 ± 0.01^a^	0.2 ± 0.01^a^	0.2 ± 0.02^a^
	*L. plantarum*	2.1 ± 0.00^bc^	2.0 ± 0.02^bc^	2.1 ± 0.01^bc^	2.1 ± 0.02^bc^	2.1 ± 0.01^bc^	0.2 ± 0.04^a^	0.2 ± 0.03^a^	0.2 ± 0.05^a^

*Results are expressed as mean ± standard deviation from the three replicates. Values with different letters indicate a significant difference (p < 0.05)*.

The content of ferulic acid was significantly decreased from around 4.7 mg/L to about 0.8 mg/L (*P* < 0.05) after storage for 10 days, which may also be related to the metabolism of ferulic acid into 4-vinyl guaiacol and other substances (43). Also, caffeic acid content increased rapidly, which could be attributed to the conversion of dihydrocaffeic acid to caffeic acid (47). Meanwhile, the content of catechin in apple juice fermented by *L. acidophilus, L. plantarum*, and *L. casei* were increased from 10.1 ± 0.1 mg/L, 9.9 ± 0.3 mg/L, 10.5 ± 0.4 mg/L to 12.6 ± 0.9 mg/L, 14.0 ± 0.5 mg/L, and 14.7 ± 0.3 mg/L while the content of protocatechuic acid were decreased from 24.4 ± 0.09 mg/L, 22.8 ± 2.6 mg/L, 25.1 ± 0.3 mg/L to 16.2 ± 0.8 mg/L, 18.9 ± 2.5 mg/L, and 18.2 ± 0.6 mg/L in the first 10-day storage. It has been reported that LAB can metabolize protocatechuic acid to catechin through the decarboxylation reaction (48). The study of Annalisa et al. also demonstrated the metabolic correlation between protocatechuic acid and catechin (49). It was also found that the changes of rutin and quercetin were almost the same during fermentation and storage at 4°C. Moreover, the contents of rutin and quercetin were almost stable throughout fermentation and there was no significant difference among the three LAB fermented samples (*P* ≥ 0.05). However, the contents of rutin and quercetin in all the samples decreased significantly after storage for 10 days *(P* < 0.05), which may be attributed to the uncoupling of beta-glucosidase in lactic acid bacteria (50). Meanwile, rutin and quercetin could be biotransformed to low molecular weight metabolites, such as 2-(3,4-dihydroxyphenyl) acetic acid, 2-(3-hydroxyphenyl) acetic acid, 3,4-dihydroxybenzoic acid and others (51). The gallic acid content was increased during 72-h fermentation, but decreased during storage at 4°C for 30 days. In contrast, the content of proanthocyanidin B_2_ was decreased rapidly within the first 12 h of fermentation and then stayed stable in the following 36 h of fermentation. Similar to gallic acid, the proanthocyanidin B_2_ content was decreased significantly during storage.

**Figure 3:**
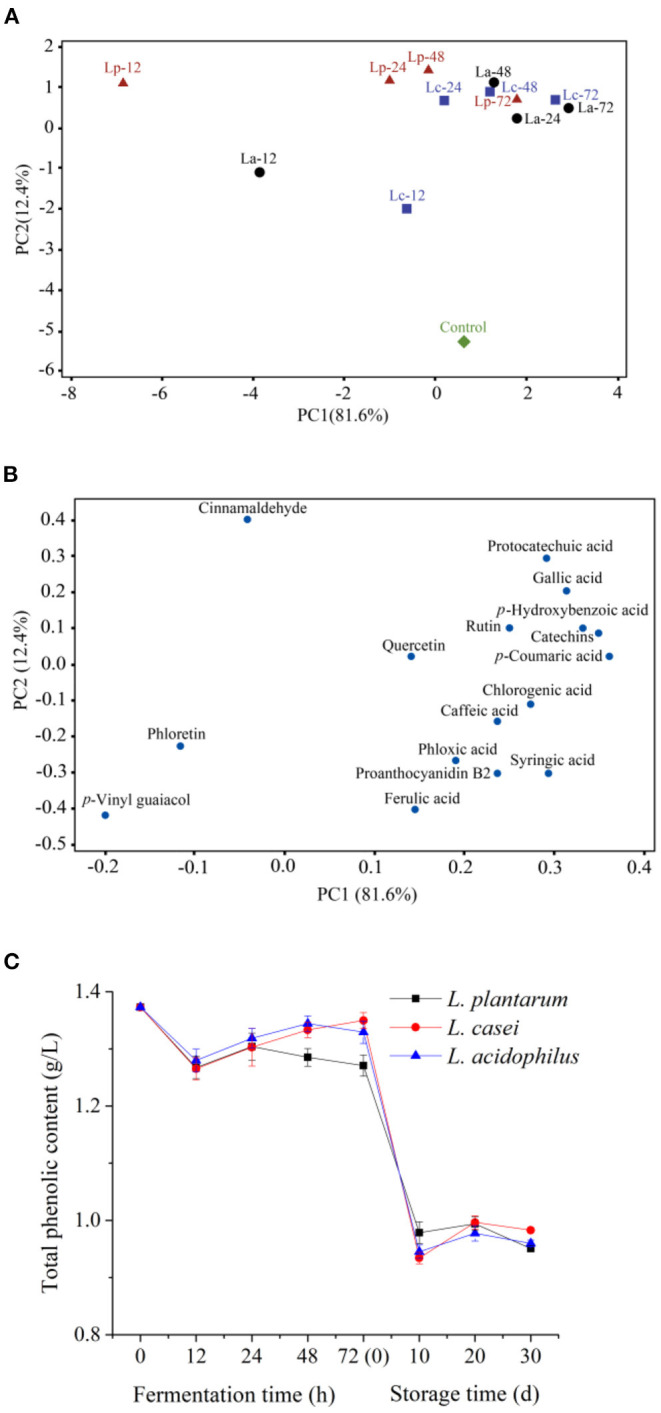
Principal component analysis of phenolic profile in apple juice during fermentation by the three LAB strains [**(A)**, the score plots; **(B)**, the loading plots] and changes in total phenolic content **(C)**. “Control,” Unfermented apple juice; “La,” Apple juice fermented by *L. acidophilus*; “Lp”, Apple juice fermented by *L. plantarum*; “Lc”, Apple juice fermented by *L. casei*. The number followed with the name of strain indicated the time of fermentation.

The changes of individual phenolic compounds during 72-h fermentation were analyzed by PCA (Figures 3A,B). PC1 and PC2 explained 94% of the total variance of all the “fuji” apple juices. According to Figure 3A, the unfermented sample was located on the positive side of PC1. And apple juices fermented with *L. acidophilus, L. plantarum*, and *L. casei* at different stage were distributed on the different PC1 region, implying that PC1 can be considered as a contributor to distinguish the unfermented and fermented samples. In the loading plot of phenolic acids (Figure 3B), three phenolics, including cinnamaldehyde, phloretin, and *p*-vinyl guaiacol, were located on the negative side of PC1. In contrast, the other 13 phenolic acids were located on the positive side of PC1. Among them, *p*-vinyl guaiacol, phloretin, cinnamaldehyde, quercetin and ferulic acid were placed nearby the origin of first PC. Therefore, LAB fermentation may have minor effects on these phenolic acids. However, protocatechuic acid, gallic acid, *p*-hydroxybenzoic acid, *p*-coumaric acid, chlorogenic acid and syringic acid were all placed far from the origin of first PC and positively correlated. This result implied that LAB fermentation has some positive effects on the phenolic acids in apple juice.

The changes in total phenolic content in apple juices fermented by the three LAB strains are shown in Figure 3C. Overall, the total phenolic content was declined after 72-h fermentation (*P* < 0.05). During the first 12 h of fermentation, the total phenolic contents of apple juice fermented with different strains all declined from 1.37 g/L to around 1.27 g/L. However, the total phenolic content in apple juice fermented by *L. casei* was increased to 1.35 ± 0.01 g/L after 72 h of fermentation (*P* < 0.05), while that in apple juice fermented by *L. plantarum* was decreased to 1.27 ± 0.02 g/L. The loss of phenolics may be due to the interaction between phenolics and proteins in apple juice during fermentation, which could produce insoluble complexes (4). Another potential reason is that phenolics are the inhibitors of the growth of *L. plantarum*, and LAB can synthetize a series of enzymes (benzyl alcohol dehydrogenase, decarboxylase, tannase) to degrade phenolics (42, 52). The total phenolic contents in apple juice fermented by the three LAB strains were close after fermentation. In the first 10 day of storage, the total phenolic content decreased continuously to about 0.95 g/L. After that, no significant change in the total phenolic content was observed in the subsequent 20-day storage. Similar results were found by Wang et al. (53), the total phenolic content of fermented mango juice experienced a rapid decrease during the first 10-d storage and then stable. This phenomenon may be caused by the presence of dissolved oxygen which resulted in the oxidation of phenolic compounds during the first 10-d storage (54). However, phenolic compounds were more stable with the consumption of oxygen during the later storage stage.

### Change in the Antioxidant and Antimicrobial Activities *in vitro* During LAB Fermentation and Storage

**Figure 4:**
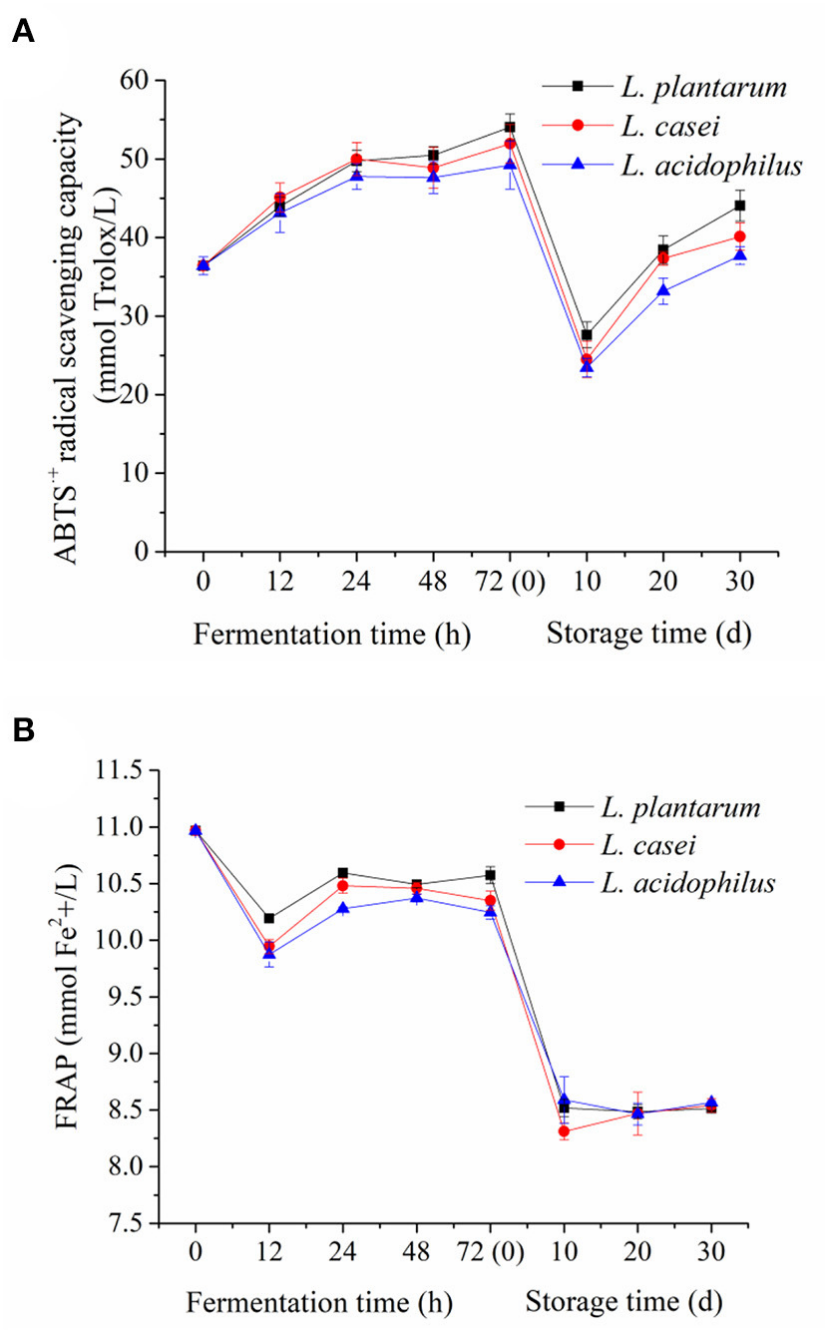
The antioxidant activity of unfermented and LAB-fermented apple juice *in vitro*. **(A)** ABTS^·+^ radical scavenging activity, **(B)** ferric reducing antioxidant power.

The antioxidant properties of apple juice are mainly related to vitamin C and phenolics (55, 56). ABTS^·+^ method (free radical scavenging capacity) and FRAP method (iron reduction capacity) were used to evaluate the effect of LAB fermentation on the antioxidant activity of apple juice *in vitro*. The results of antioxidant activity of fermented apple juice are shown in Figure 4. Generally, LAB fermentation enhanced the ABTS^·+^ radical scavenging capacity of the apple juice fermented by *L. acidophilus, L. plantarum*, and *L. casei* were increased from 36.41 ± 1.1 mmol Trolox/L to 49.22 ± 3.0 mmol Trolox/L, 54.03 ± 1.7 mmol Trolox/L, 51.94 ± 2.4 mmol Trolox/L, which was consistence with the trend in pomegranate juice (57) and jussara pulp (58) fermented by LAB. On the other hand, both ABTS^·+^ radical scavenging capacity and ferric reducing power of fermented apple juice declined significantly during the first 10-day storage, which could be related to the aforementioned decrease of total phenolic content in fermented apple juice. In the next 20-day storage, their trends have different performance. The ferric reducing power was stable, whereas the ABTS^·+^ radical scavenging capacity was even increased. Overall, the ferric reducing ability of fermented apple juice has similar trend with the change of total phenolic acid content whereas the ABTS^·+^ radical scavenging capacity of fermented apple juice were increased during fermentation. Such inconsistent changes in ferric reducing ability and ABTS^·+^ radical scavenging capacity of fermented juice were also found by Wang et al. (9) who used LAB to ferment apple juice. There are two possible reasons for this phenomenon. First one is that the dissolved oxygen and microbial strains can affect the ABTS^·+^ radical scavenging capacity of fermented apple juice (9). Another one is that the bioconversion of phenolic acids during fermentation (59). Ozgen et al. (60) compared the ABTS^·+^ radical scavenging activities of four phenolic acids (gallic acid, chlorogenic acid, caffeic acid and ascorbic acid), and reported that gallic acid had the highest ABTS^·+^ radical scavenging activities, followed by caffeic acid. Zhang et al. (61) illustrated that gallic acid and caffeic acid had more contribution to the ABTS^·+^ radical scavenging activity as compared to ferulic acid. And in our study, we also found an increase in these phenolic acid contents (gallic acid, caffeic acid and so on).

**Figure 5:**
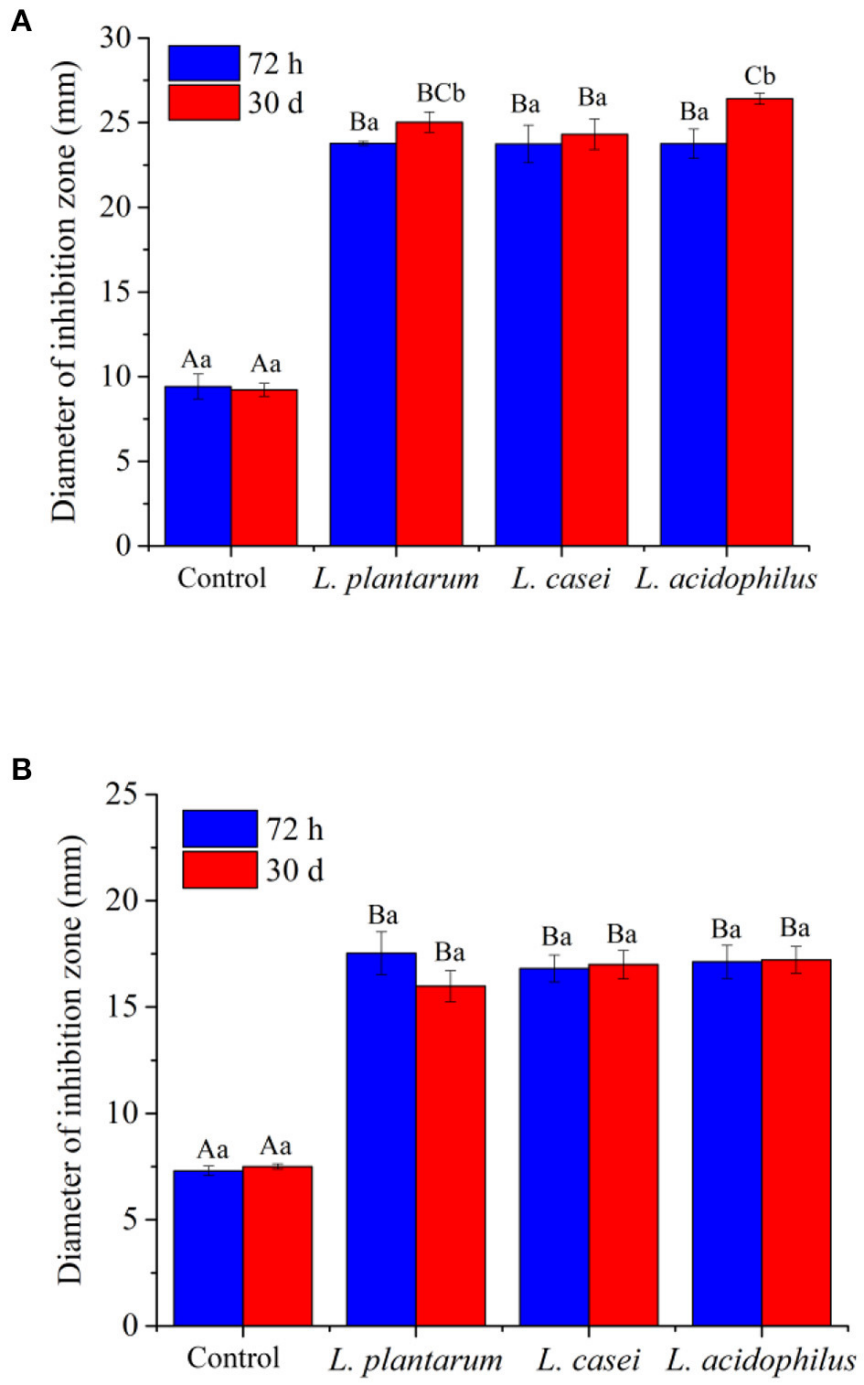
The antimicrobial activity of unfermented and LAB-fermented apple juice against the growths of **(A)** *E. coli* and **(B)** *S. aureus in vitro*. Values with different letters within each samples indicated a significant difference in content (*p* < 0.05).

In addition, microbial contamination is an important reason of food spoilage. *E. coli* (G^−^) and *S. aureus* (G^+^) are the most common microorganisms in contaminated food (62). In this study, the antimicrobial activities of unfermented and fermented apple juice against the growth of *E. coli* and *S. aureus* were evaluated, and the results are shown in Figure 5. Before fermentation, the samples had weak antibacterial activities. After 72-h fermentation, the diameters of the inhibition circles against *E. coli* and *S. aureus* were 23.78 ± 0.1 mm and 17.53 ± 1.0 mm for the apple juice fermented by *L. plantarum*, 23.74 ± 1.1 mm and 16.81 ± 0.6 mm for the juice fermented by *L. casei*, and 23.76 ± 0.9 mm and 17.13 ± 0.8 mm for the juice fermented by *L. acidophilus*. It is supposed that the antibacterial properties of fermented apple juice may be related to bacteriocin, diacetyl, organic acid and hydrogen peroxide produced by lactic acid bacteria metabolism (63). Although LAB fermentation improved the antimicrobial activities, there was no significant difference in the areas of inhibition zone of apple juice fermented by the three LAB strains after 72-h fermentation. Interestingly, after storage for 30 days, the diameter of the inhibition circle against *E. coli* for all the juices even increased. The apple juice fermented with *L. acidophilus* had the largest inhibition circles (26.41 ± 0.3 mm) followed by those fermented with *L. plantarum* (25.03 ± 0.6 mm) and *L. casei* (24.31 ± 0.9 mm). In addition, the diameter of the inhibition circle against *S. aureus* for the samples fermented with *L. acidophilus* did not change significantly after 30-day storage, whereas the antimicrobial ability of samples fermented with *L. casei* and *L. plantarum* against *S. aureus* got weakened with storage. Generally, the antimicrobial activities of unfermented and fermented apple juice can be contributed by the acid production, these acids can lower pH and create an unfavorable environment for pathogens and deteriorating microorganisms (64). Additionally, LAB can produce some antimicrobial compounds such as H_2_O_2_ and bacteriocin, which can inhibit microbial growth (21). Therefore, such changes in antimicrobial activities after fermentation and storage may be influenced by the change of organic acids and viable cell counts of LAB.

## Conclusion

In this study, three commercial LAB strains including *L. acidophilus, L. casei*, and *L. plantarum* were utilized to ferment apple juice. All the microbial strains presented satisfactory growth capacities in the studied apple juice environment. In general, LAB fermentation improved the antioxidant and antimicrobial capacities by metabolizing phenolics and producing lactic acid. In addition, the changes in the physicochemical properties of fermented apple juice during storage at 4°C for 30 days was also evaluated. The results showed that the total amino acid content was significantly increased, but the total phenolic content was significantly decreased. Overall, these results prove that lactic acid fermentation is a useful method to enhance the nutritional value of apple juice.

## Data Availability Statement

The original contributions presented in the study are included in the article/Supplementary Material, further inquiries can be directed to the corresponding author/s.

## Author Contributions

JY performed the experiments, analyzed the data, and wrote the manuscript. YS, TG, and YW analyzed the data and wrote the manuscript. HS, QZ, and CL analyzed and discussed the data. CZ, YH, and YT provided samples and discussed the data. YT designed the research content, analyzed the data, and modified the manuscript. All authors read and approved the final manuscript.

## Funding

This work was financially supported by grants from the National Natural Science Foundation of China (No. 32100037), Natural Science Research General Project of Jiangsu Higher Education Institutions (No. 20KJB550008), Science and Technology Program of Kizilsu Kirghiz Autonomous Prefecture in 2021 (No. 3-25), Jiangsu Planned Projects for Postdoctoral Research Funds (No. 2019K015), Priority Academic Program Development of Jiangsu Higher Education Institutions (PAPD), and the Research and Practice Innovation Program for College Graduates of Jiangsu Province (No. KYCX20_2888).

## Conflict of Interest

The author YT had previously collaborated with the reviewer KWC. The remaining authors declare that the research was conducted in the absence of any commercial or financial relationships that could be construed as a potential conflict of interest.

## Publisher's Note

All claims expressed in this article are solely those of the authors and do not necessarily represent those of their affiliated organizations, or those of the publisher, the editors and the reviewers. Any product that may be evaluated in this article, or claim that may be made by its manufacturer, is not guaranteed or endorsed by the publisher.

## Correction Note

A correction has been made to this article. Details can be found at: 10.3389/fnut.2025.1695110.
