# Expression of Concern: Fermentation and storage characteristics of “fuji” apple juice using *Lactobacillus acidophilus, Lactobacillus casei* and *Lactobacillus plantarum*: microbial growth, metabolism of bioactives and *in vitro* bioactivities

**DOI:** 10.3389/fnut.2024.1450420

**Published:** 2024-07-03

**Authors:** Frontiers Editorial Office

With this notice, Frontiers states its awareness of serious allegations of undisclosed conflicts of interest surrounding the article “Fermentation and Storage Characteristics of “Fuji” Apple Juice Using *Lactobacillus acidophilus, Lactobacillus casei* and *Lactobacillus plantarum*: Microbial Growth, Metabolism of Bioactives and *in vitro* Bioactivities” published on 9 February 2022. Our Research Integrity team will conduct an investigation in full accordance with our procedures. The situation will be updated as soon as the investigation is complete.

