# Correction: Fermentation and storage characteristics of “Fuji” apple juice using *Lactobacillus acidophilus, Lactobacillus casei* and *Lactobacillus plantarum*: microbial growth, metabolism of bioactives and *in vitro* bioactivities

**DOI:** 10.3389/fnut.2025.1695110

**Published:** 2025-09-09

**Authors:** Frontiers Production Office

**Affiliations:** Frontiers Media SA, Lausanne, Switzerland

**Keywords:** lactic acid bacteria, apple juice, fermentation, physicochemical properties, bioactivities

The conflict of interest statement was erroneously given as “The authors declare that the research was conducted in the absence of any commercial or financial relationships that could be construed as a potential conflict of interest.”

The correct conflict of interest statement is

“The author YT had previously collaborated with the reviewer KWC.

The remaining authors declare that the research was conducted in the absence of any commercial or financial relationships that could be construed as a potential conflict of interest.”

The original version of this article has been updated.

