# Supplementary material for: Fermentation and Storage Characteristics of “Fuji” Apple Juice Using Lactobacillus acidophilus, Lactobacillus casei and Lactobacillus plantarum: Microbial Growth, Metabolism of Bioactives and in vitro Bioactivities
Source: Front Nutr. 2022 Feb 9;9:833906. doi: 10.3389/fnut.2022.833906 (PMC8864132; doi:10.3389/fnut.2022.833906)
Supplement: Supplementary file 1 [file Table_1.DOCX]

**Fermentation and Storage Characteristics of ‘Fuji’ Apple Juice using *Lactobacillus acidophilus*, *Lactobacillus casei* and *Lactobacillus plantarum*: Microbial Growth, Metabolism of Bioactives and *in vitro* Bioactivities**

Jie Yang^1, 2^, Yue Sun^1, 2^, Tengqi Gao^1, 2^, Yue Wu^3^, Hao Sun^1, 2^, Qingzheng Zhu^1, 2^, Chunsheng Liu^1, 2^, Chuang Zhou^4^, Yongbin Han^5^, Yang Tao^5*^

^1^Jiangsu Key Laboratory of Marine Bioresources and Environment/Jiangsu Key Laboratory of Marine Biotechnology, Jiangsu Ocean University, Lianyungang, 222005, China

^2^Co-Innovation Center of Jiangsu Marine Bio-industry Technology, Jiangsu Ocean University, Lianyungang 222005, China

^3^Sonochemistry Group, School of Chemistry, The University of Melbourne, Parkville, Victoria 3010, Australia

^4^Department of Animal Husbandry and Veterinary Medicine, Jiangsu Vocational College of Agriculture and Forestry, Jurong 212400, China

^5^College of Food Science and Technology, Nanjing Agricultural University, 1 Weigang, Nanjing 210095, China

*Corresponding authors:

Yang Tao, Email: yang.tao@njau.edu.cn

College of Food Science and Technology, Nanjing Agricultural University, 1 Weigang, Nanjing 210095, China

**TABLE S1** Changes in the contents of individual organic acids and sugars (mg/L) in apple juices during lactic acid fermentation and the subsequent refrigerated storage period (4 °C, 30 days).

| Compounds | strains | Time | | | | | | | |
| --- | --- | --- | --- | --- | --- | --- | --- | --- | --- |
|  |  | Fermentation for 0h | Fermentation for 12h | Fermentation for 24h | Fermentation for 48h | Fermentation for 72h (Storage for 0d) | Storage for 10d | Storage for 20d | Storage for 30d |
| Oxalate | *L. acidophilus* | 324.6±2.2^Ba^ | 396.0±2.8^Aa^ | 312.9±4.7^Ca^ | 252.5±2.0^DEa^ | 242.1±0.6^EFa^ | 239.5±7.16^Fa^ | 250.6±5.9^Ea^ | 261.3±2.8^Da^ |
|  | *L. casei* | 324.6±2.2^Ba^ | 472.6±100.7^Aa^ | 310.8±2.5^BCa^ | 254.8±8.3^BCa^ | 243.9±1.8^Ca^ | 248.7±8.2^BCa^ | 253.8±4.6^BCa^ | 261.6±2.2^BCa^ |
|  | *L. plantarum* | 324.6±2.2^Ba^ | 396.6±4.1 ^Aa^ | 302.8±1.8^Ea^ | 250.5±1.6^Ea^ | 241.6±0.4^Fa^ | 240.3±2.4^Fa^ | 248.9±2.9^EGa^ | 258.2±1.9^Da^ |
| Pyruvic acid | *L. acidophilus* | 96.2±0.9^Ba^ | 82.6±0.7^BCa^ | 126.0±1.0^Aa^ | 74.3±1.3^BCa^ | 74.9±0.3^BCa^ | 90.9±16.4^BCa^ | 88.8±25.6^BCa^ | 71.8±0.2^Ca^ |
|  | *L. casei* | 96.2±0.9 ^Ba^ | 96.1±1.7^Bb^ | 121.5±1.8^Ab^ | 74.0±1.6^BCa^ | 74.6±0.6^BCa^ | 85.7±25.5^BCa^ | 79.3±18.8^BCa^ | 70.4±0.8^Cab^ |
|  | *L. plantarum* | 96.2±0.9 ^Ba^ | 85.5±1.1^BCb^ | 130.2±1.4^Ac^ | 74.9±0.3^Ca^ | 75.2±0.3 ^Ca^ | 74.5±1.7^Ca^ | 78.6±17.0^Ca^ | 72.6±0.8^Cb^ |
| Malic acid | *L. acidophilus* | 1166.5±14.7^Aa^ | 85.3±3.8^Bb^ | 33.9±1.2^Db^ | 28.9±2.9^Ea^ | 45.9±0.4^Ca^ | 38.3±2.5^Da^ | 35.5±1.8^Da^ | 48.6±0.7^Dab^ |
|  | *L. casei* | 1166.5±14.7^Aa^ | 98.3±2.1^Ba^ | 30.5±2.3^Eb^ | 32.1±2.5^Ea^ | 47.9±0.9 ^Ca^ | 31.8±2.4^Ea^ | 38.8±3.3^Da^ | 50.5±0.7^Ca^ |
|  | *L. plantarum* | 1166.5±14.7^Aa^ | 87.9±2.2 ^Bb^ | 39.2±2.3^Da^ | 27.9±0.5^Ea^ | 42.9±2.5^CDb^ | 36.2±4.4^Da^ | 35.6±0.9^Da^ | 46.5±1.1^Cb^ |
| Lactic acid | *L. acidophilus* | 0^Aa^ | 1422.6±14.3^Ba^ | 2230.2±11.7^Cb^ | 3437.6±52.7^Da^ | 4178.7±19.2^Ea^ | 4727.4±26.0^Fab^ | 4657.9±256.4^Fa^ | 4781.8±5.6^Fbc^ |
|  | *L. casei* | 0^Aa^ | 1307.6±110.0^Bb^ | 2264.5±10.9^Ca^ | 3500.8±48.0 ^Da^ | 4191.6±31.6^Ea^ | 4668.1±40.3^Fb^ | 4847.8±45.7^Ga^ | 4773.9±15.5^Gc^ |
|  | *L. plantarum* | 0^Aa^ | 1490.5±8.0 ^Ba^ | 2242.8±12.7^Cab^ | 3406.6±100.0 ^Da^ | 4148.1±15.9^Ea^ | 4775.8±37.6^Ga^ | 4658.5±46.2 ^Fa^ | 4845.4±19.9^Ga^ |
| Citric acid | *L. acidophilus* | 730.7±13.1^Aa^ | 333.9±65.1^Ba^ | 312.7±8.0^Ba^ | 9.5±1.0^Ca^ | 10.9±9.5^Ca^ | 0^Ca^ | 0^Ca^ | 0^Ca^ |
|  | *L. casei* | 730.7±13.1^Aa^ | 504.4±280.1^Ba^ | 279.3±3.1^Cc^ | 8.1±0.5^Da^ | 4.3±7.4^Da^ | 0^Da^ | 0^Da^ | 0^Da^ |
|  | *L. plantarum* | 730.7±13.1^Aa^ | 353.7±1.5^Ba^ | 297.7±3.2^Cb^ | 10.9±4.1^Da^ | 5.1±8.9^Dea^ | 3.6±3.1^Ea^ | 0^Ea^ | 1.2±2.1^Ea^ |
| Succinic acid | *L. acidophilus* | 160.7±8.3^Aa^ | 113.6±14.9^Ca^ | 144.7±25.2^ABb^ | 143.6±7.5^ABa^ | 136.0±1.1^Bb^ | 65.0±16.6^Da^ | 45.0±3.0^Db^ | 50.0±2.2^Da^ |
|  | *L. casei* | 160.7±8.3^Aa^ | 175.2±99.8^Aa^ | 145.2±7.3^Ab^ | 146.4±1.9^Aa^ | 133.2±3.5^Ab^ | 59.5±3.9^Ba^ | 44.0±4.2^Bb^ | 46.8±1.7^Ba^ |
|  | *L. plantarum* | 160.7±8.3^Aa^ | 101.9±12.3^Ba^ | 181.0±12.2^Aa^ | 124.6±7.2^ABb^ | 148.2±3.2^ABa^ | 50.2±3.5^Ca^ | 56.4±7.9^Ca^ | 50.4±2.7^Ca^ |
| Fructose | *L. acidophilus* | 52.0±2.3^Abc^ | 49.1±3.2^Ac^ | 49.8±2.4^ABc^ | 50.8±3.1^Abc^ | 49.4±5.4^Ac^ | 55.9±0.8^Aab^ | 53.7±0.8^Cabc^ | 58.2±0.8^Aa^ |
|  | *L. casei* | 52.0±2.3^Aabc^ | 52.8±4.5^Aab^ | 46.8±2.0^Bd^ | 49.6±3.6^Abcd^ | 47.6±3.2^Acd^ | 56.5±1.9^Aa^ | 56.0±1.0^Ba^ | 56.6±1.2^Aa^ |
|  | *L. plantarum* | 52.0±2.3^Ac^ | 48.4±2.4^Ad^ | 51.1±1.0^Acd^ | 50.7±0.9^Acd^ | 49.1±1.2^Acd^ | 55.3±1.4^Ab^ | 58.4±0.4^Aa^ | 57.2±1.8^Aab^ |
| Sorbitol | *L. acidophilus* | 5.1±0.3^Aab^ | 4.6±0.3^Abcd^ | 4.5±0.2^Acd^ | 4.5±0.3^Acd^ | 4.4±0.5^Ad^ | 4.9±0.1^Aabc^ | 4.6±0.1^Bbcd^ | 5.3±0.2^Aa^ |
|  | *L. casei* | 5.1±0.3^Aab^ | 5.0±0.4^Aab^ | 4.2±0.2^Bd^ | 4.4±0.2^Acd^ | 4.1±0.2^Ad^ | 4.8±0.2^ABbc^ | 4.8±0^ABbc^ | 5.3±0.1^Aa^ |
|  | *L. plantarum* | 5.1±0.3^Aa^ | 4.6±0.2^Abcd^ | 4.6±0.1^Abc^ | 4.5±0.1^Acd^ | 4.3±0.1^Ad^ | 4.5±0.1^Bcd^ | 4.9±0.2^Aab^ | 5.0±0.1^Aa^ |
| Glucose | *L. acidophilus* | 17.7±0.8^Aa^ | 16.5±0.4^Ab^ | 13.9±0.4^Acd^ | 12.7±0.4^Ae^ | 12.1±0.4^Ae^ | 13.5±0.1^Ad^ | 12.7±0.1^Be^ | 14.3±0.2^Ac^ |
|  | *L. casei* | 17.7±0.8^Aa^ | 16.9±1.5^Aa^ | 14.2±1.4^Ab^ | 12.6±0.9^Abc^ | 11.5±0.6^Ac^ | 13.2±0.3^Ab^ | 13.5±0.1^Ab^ | 14.2±0.1^Ab^ |
|  | *L. plantarum* | 17.7±0.8^Aa^ | 15.1±0.6^Ab^ | 14.2±0.4^Ac^ | 12.7±0.2^Ad^ | 11.7±0.1^Ae^ | 12.7±0.1^Bd^ | 13.5±0.1^Ac^ | 14.2±0.1^Ac^ |
| Sucrose | *L. acidophilus* | 21.4±0.2^Abcd^ | 22.3±0.6^Abc^ | 21.0±2.2^Acd^ | 20.8±0.1^Acd^ | 19.9±0.8^Ad^ | 22.7±0.2^Aab^ | 21.3±0.1^Cbcd^ | 23.8±0^Aa^ |
|  | *L. casei* | 21.4±0.2^Ab^ | 21.7±1.3^Ab^ | 19.2±0.7^Ac^ | 19.9±1.3^Ac^ | 19.8±0.1^Ac^ | 22.0±0.5^ABb^ | 22.4±0.3^Bb^ | 23.9±0.2^Aa^ |
|  | *L. plantarum* | 21.4±0.2^Ab^ | 21.3±0.5^Ab^ | 20.6±0.2^Ac^ | 20.1±0.4^Ac^ | 19.2±0.4^Ad^ | 21.5±0.3^Bb^ | 23.4±0.2^Aa^ | 23.7±0.2^Aa^ |

Results are expressed as mean ± standard deviation from the three replicates.

Values with different letters indicate a significant difference (*p* < 0.05).
